# An Inflammation–renal composite approach to predict contrast-induced nephropathy in acute myocardial infarction

**DOI:** 10.1016/j.ijcrp.2026.200672

**Published:** 2026-07-02

**Authors:** Ahmet Ferhat Kaya, Görkem Ayhan, Veysi Can, Emrah Özbek, Ömer Kümet

**Affiliations:** Department of Cardiology, Van Regional Education and Research Hospital, Van, Turkey

**Keywords:** Acute myocardial infarction, Contrast-induced nephropathy, Percutaneous coronary intervention, Risk prediction, Inflammation, Cardiorenal interaction

## Abstract

**Background:**

Contrast-induced nephropathy (CIN) remains an important complication in patients with acute myocardial infarction (MI) undergoing percutaneous coronary intervention (PCI) and is associated with adverse clinical outcomes. Both systemic inflammation and baseline renal dysfunction may contribute to susceptibility to contrast-related renal injury. We aimed to develop and internally validate an inflammation–renal composite score for predicting CIN and to examine the association between CIN and in-hospital mortality.

**Methods:**

In this retrospective cohort study, 1286 consecutive patients with STEMI or NSTEMI undergoing PCI were included. CIN was defined as an increase in serum creatinine ≥0.5 mg/dL or ≥25% within 48–72 h after contrast exposure. An inflammation–renal composite (IRIS) score integrating inflammatory and renal parameters was derived using penalized regression. Model discrimination and calibration were evaluated, and the incremental predictive value beyond a clinical model was assessed.

**Results:**

CIN occurred in 427 patients (33.2%). In multivariable analysis, age (OR 1.03 per year, 95% CI 1.02–1.04), chronic kidney disease (OR 5.05, 95% CI 2.64–9.63), and the IRIS score (OR 2.72 per standard deviation increase, 95% CI 2.16–3.42) were independently associated with CIN. Incorporation of the IRIS score resulted in a modest improvement in model discrimination (AUC 0.686 vs. 0.698). CIN was independently associated with increased in-hospital mortality (OR 3.35, 95% CI 1.58–7.11).

**Conclusion:**

In patients with acute MI undergoing PCI, CIN was common and independently associated with in-hospital mortality. The IRIS score, derived from routinely available inflammatory and renal parameters, was independently associated with CIN and may represent a practical bedside tool for risk stratification before PCI. Prospective multicenter studies are warranted to externally validate its clinical utility.

## 1 Introduction

Acute myocardial infarction (MI) remains a major cause of morbidity and mortality worldwide despite substantial advances in pharmacological therapies and percutaneous coronary intervention (PCI) techniques [1,2]. Early risk stratification in patients undergoing PCI is essential for identifying complications that may adversely affect short-term outcomes.

Contrast-induced nephropathy (CIN), also referred to as contrast-associated acute kidney injury, is one of the most frequent non-cardiac complications following coronary angiography and PCI. Although the use of lower-osmolar contrast media and preventive strategies has reduced its incidence, CIN remains associated with prolonged hospitalization, increased healthcare costs, and higher mortality [[3], [4], [5]]. Patients presenting with acute coronary syndromes may be particularly vulnerable due to hemodynamic instability, systemic inflammation, and the urgent nature of revascularization procedures.

The pathophysiology of CIN is multifactorial and involves renal medullary hypoxia, oxidative stress, endothelial dysfunction, and direct tubular toxicity [6,7]. In addition to these mechanisms, systemic inflammatory activation has been increasingly recognized as a potential contributor to contrast-related renal injury. In acute MI, inflammatory pathways are amplified by plaque rupture and ischemia–reperfusion processes, which may influence both cardiovascular and renal function [[8], [9], [10]]. Therefore, renal vulnerability in MI may not be fully explained by baseline kidney function alone.

Several studies have investigated inflammation-related biomarkers—including the neutrophil-to-lymphocyte ratio, systemic immune-inflammation index, and C-reactive protein–based parameters—in relation to cardiovascular outcomes and CIN [[11], [12], [13], [14]]. However, most previous investigations have evaluated these markers individually. Approaches that integrate inflammatory markers with objective measures of renal function have been less frequently explored, particularly in contemporary cohorts of patients with both STEMI and NSTEMI undergoing PCI [15,16].

Given the close interaction between inflammation and renal dysfunction in acute MI, a combined approach incorporating both domains may provide additional insight into CIN risk stratification. However, whether such an integrated strategy offers incremental predictive value beyond conventional clinical variables remains uncertain.

Therefore, the present study aimed to develop and internally validate an inflammation–renal composite score for predicting contrast-induced nephropathy in patients with acute MI undergoing PCI. In addition, we evaluated the association between CIN and in-hospital mortality.

## 2 Results

### 2.1 Study population

A total of 1286 patients with acute myocardial infarction (including both STEMI and NSTEMI) who underwent PCI were included in the analysis. Contrast-induced nephropathy (CIN) occurred in 427 patients (33.2%). In-hospital mortality was observed in 31 patients (2.4%).

### 2.2 Baseline characteristics

Baseline clinical, renal, and laboratory characteristics according to CIN status are presented in Table 1. Patients who developed CIN were older (65 [58–72] vs. 63 [54–70] years, p < 0.001) and more frequently male (49.4% vs. 39.7%, p < 0.001). Chronic obstructive pulmonary disease was more prevalent in the CIN group (20.6% vs. 12.6%, p < 0.001), whereas the prevalence of diabetes mellitus, hypertension, and prior coronary artery disease did not differ significantly between groups.

**Table 1:** Baseline characteristics of the study population stratified by contrast-induced nephropathy.

Variable	CIN (−) (n = 859)	CIN (+) (n = 427)	P value
Age (years)	63 (54–70)	65 (58–72)	<0.001
Male sex	341 (39.7%)	211 (49.4%)	<0.001
Diabetes mellitus	325 (37.8%)	177 (41.5%)	0.210
Hypertension	418 (48.7%)	200 (46.8%)	0.538
Chronic kidney disease	43 (5.0%)	27 (6.3%)	0.327
COPD	108 (12.6%)	88 (20.6%)	<0.001
Smoking	343 (39.9%)	180 (42.2%)	0.444
Prior CAD	297 (34.6%)	135 (31.6%)	0.290
Hyperlipidemia	268 (31.2%)	121 (28.3%)	0.293
Multivessel disease	267 (31.1%)	109 (25.5%)	0.039
Left ventricular ejection fraction (%)	60 (50–60)	60 (50–60)	0.388
Systolic blood pressure (mmHg)	125 (120–140)	130 (120–140)	0.088
NYHA functional class	1 (1–1)	1 (1–1)	0.002
Creatinine (mg/dL)	0.80 (0.68–0.9)	0.90 (0.75–1.07)	<0.001
Creatinine at 72 h (mg/dL)	0.90 (0.80–1.10)	1.10 (0.90–1.24)	<0.001
eGFR (mL/min/1.73m^2^)	88.20 (72.50–101.00)	84.55 (67.39–97.50)	<0.001
Hemoglobin (g/dL)	14.00 (12.80–15.20)	13.90 (12.45–14.90)	<0.001
WBC (×10^3^/μL)	10.09 (8.20–12.10)	9.90 (7.95–11.49)	0.104
CRP (mg/L)	3.16 (1.20–8.31)	2.38 (0.80–8.30)	0.002
Albumin (g/dL)	4.20 (3.29–4.85)	4.20 (3.55–4.80)	0.075
NLR	3.33 (2.37–5.74)	3.24 (2.14–5.20)	0.152
SII	773.17 (513.81–1471.43)	785.48 (496.72–1295.38)	0.780
CAR	0.53 (0.18–1.32)	0.29 (0.11–1.48)	<0.001
PIV	438.39 (282.08–785.86)	432.00 (235.99–730.37)	0.185
Troponin	18.86 (2.48–105.58)	36.14 (5.79–300.00)	<0.001

Continuous variables are presented as median (interquartile range).

Categorical variables are presented as number (percentage).

P values were calculated using the Mann–Whitney *U* test for continuous variables and the χ^2^ test or Fisher's exact test for categorical variables, as appropriate.

eGFR was calculated using the CKD-EPI equation.

CIN was defined as an increase in serum creatinine ≥0.5 mg/dL or ≥25% from baseline within 48–72 h after contrast exposure.

Abbreviations: CAD, coronary artery disease; COPD, chronic obstructive pulmonary disease; eGFR, estimated glomerular filtration rate; EF, ejection fraction; NLR, neutrophil-to-lymphocyte ratio; SII, systemic immune-inflammation index; CAR, C-reactive protein-to-albumin ratio; PIV, pan-immune-inflammation value; SBP, systolic blood pressure.

Regarding renal parameters, baseline serum creatinine levels were higher in patients with CIN (0.90 [0.75–1.07] vs. 0.80 [0.68–0.90] mg/dL, p < 0.001), and estimated glomerular filtration rate was lower (84.55 [67.39–97.50] vs. 88.20 [72.50–101.00] mL/min/1.73 m^2^, p < 0.001). As expected, creatinine levels at 72 h were significantly higher among patients who developed CIN (p < 0.001).

Among laboratory parameters, hemoglobin levels were lower in the CIN group (p < 0.001), and troponin concentrations were higher (36.14 [5.79–300.00] vs. 18.86 [2.48–105.58], p < 0.001). Although C-reactive protein and the C-reactive protein–to–albumin ratio differed between groups, inflammation-derived indices such as NLR, SII, and PIV were not significantly different.

### 2.3 Multivariable predictors of CIN

Multivariable logistic regression analysis is presented in Table 2. After adjustment for clinically relevant covariates, age (OR 1.03 per year, 95% CI 1.02–1.04, p < 0.001), male sex (OR 1.30, 95% CI 1.01–1.66, p = 0.043), and chronic kidney disease (OR 5.05, 95% CI 2.64–9.63, p < 0.001) were independently associated with CIN.

**Table 2:** Multivariable logistic regression analysis for predictors of contrast-induced nephropathy.

Predictor	Adjusted OR (95% CI)	P value
Age (per year)	1.03 (1.02–1.04)	<0.001
Male sex	1.30 (1.01–1.66)	0.043
Diabetes mellitus	1.12 (0.86–1.44)	0.400
Hypertension	0.82 (0.64–1.06)	0.125
Chronic kidney disease	5.05 (2.64–9.63)	<0.001
Left ventricular ejection fraction (%)	0.99 (0.97–1.00)	0.080
Systolic blood pressure (mmHg)	1.00 (1.00–1.01)	0.325
Hemoglobin (g/dL)	0.95 (0.89–1.02)	0.150
**IRIS score (per SD increase)**	**2.72 (2.16–3.42)**	**<0.001**

Adjusted for age, sex, diabetes mellitus, hypertension, chronic kidney disease, left ventricular ejection fraction, systolic blood pressure, and hemoglobin.

IRIS score was modeled as a continuous variable standardized to one standard deviation.

Model discrimination (AUC): 0.698.

The inflammation–renal composite (IRIS) score was also independently associated with CIN (OR 2.72 per standard deviation increase, 95% CI 2.16–3.42, p < 0.001).

### 2.4 Model performance

Addition of the IRIS score to the clinical model resulted in a modest increase in discrimination. The area under the curve improved from 0.686 (95% CI 0.656–0.716) for the clinical model to 0.698 (95% CI 0.668–0.727) for the inflammation–renal model (Table 3).

**Table 3:** Model performance for prediction of contrast-induced nephropathy.

Model	AUC (95% CI)	Brier score	ΔAUC	P for comparison
Clinical model	0.686 (0.656–0.716)	0.202	—	—
Inflammation–renal model	0.698 (0.668–0.727)	0.199	0.012	0.08^a^

a P value calculated using DeLong test.

**Fig. 1:**
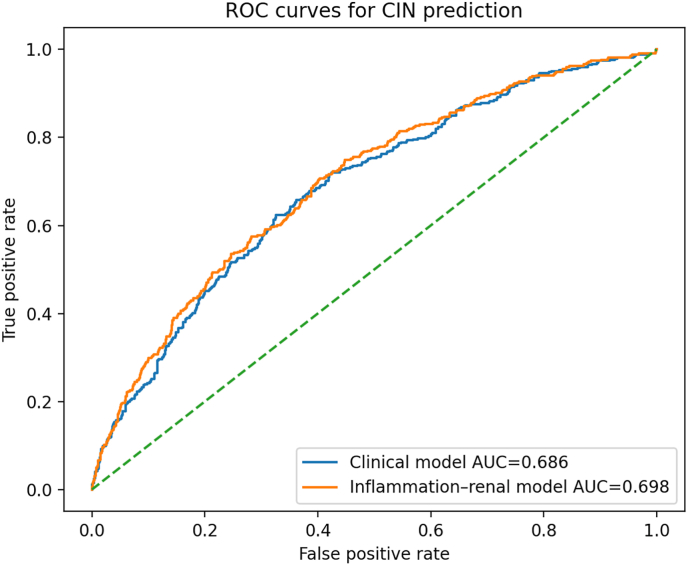
Receiver operating characteristic (ROC) curves for prediction of contrast-induced nephropathy ROC curves comparing the clinical model and the inflammation–renal composite model. AUC values are presented for each model.

**Fig. 2:**
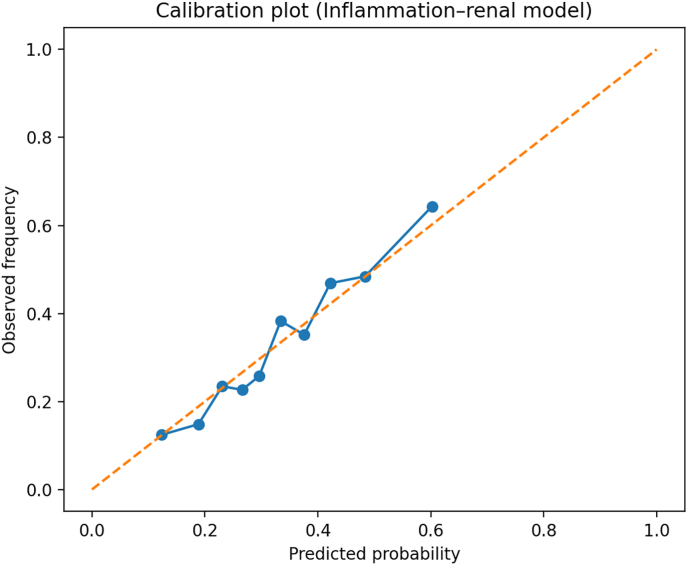
Calibration plot of the inflammation–renal composite model Calibration curve showing agreement between predicted and observed risk of contrast-induced nephropathy across deciles of predicted probability.

**Fig. 3:**
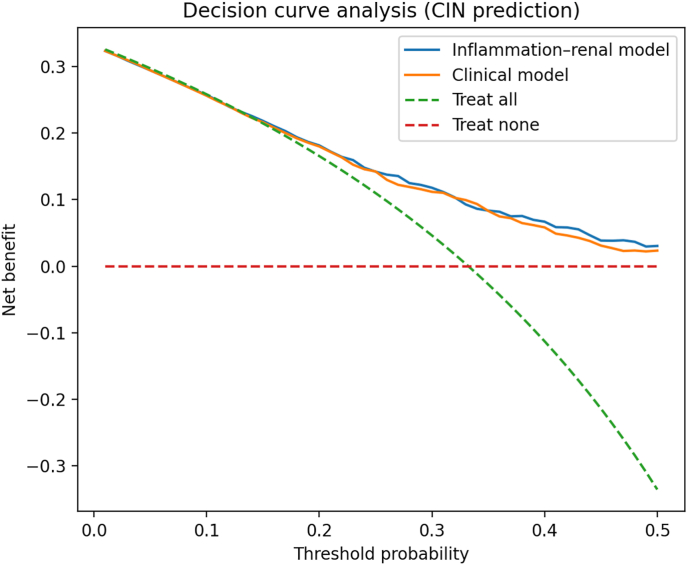
Decision curve analysis for prediction of contrast-induced nephropathy Decision curve analysis demonstrating net benefit of the clinical model and the inflammation–renal composite model across a range of threshold probabilities.

The Brier score showed a slight improvement (0.202 vs. 0.199). Calibration analysis demonstrated acceptable agreement between predicted and observed risk. Decision curve analysis indicated a numerically higher net benefit of the inflammation–renal model across a range of clinically relevant threshold probabilities (Fig. 1, Fig. 2, Fig. 3).

### 2.5 CIN and in-hospital mortality

During hospitalization, mortality occurred more frequently among patients who developed CIN. In multivariable analysis adjusted for age, chronic kidney disease, and left ventricular ejection fraction, CIN remained independently associated with in-hospital mortality (OR 3.35, 95% CI 1.58–7.11, p = 0.002) (Table 4).

**Table 4:** Multivariable logistic regression analysis for in-hospital mortality.

Predictor	Adjusted OR (95% CI)	P value
Contrast-induced nephropathy	3.35 (1.58–7.11)	0.002
Age (per year)	1.06 (1.02–1.09)	0.002
Chronic kidney disease	1.03 (0.28–3.82)	0.962
Left ventricular ejection fraction (%)	0.97 (0.93–1.00)	0.064

Adjusted for age, chronic kidney disease, and left ventricular ejection fraction.

Due to the limited number of in-hospital deaths, the model was restricted to clinically relevant covariates to maintain an appropriate events-per-variable ratio.

## 3 Discussion

In this cohort of patients with acute myocardial infarction undergoing PCI, contrast-induced nephropathy occurred in approximately one-third of the population and was independently associated with in-hospital mortality. In addition to established determinants such as age and chronic kidney disease, an inflammation–renal composite score was independently associated with CIN.

CIN remains a clinically important complication in contemporary PCI practice [17,18]. Despite improvements in contrast agents and preventive strategies, contrast-associated kidney injury continues to occur frequently in patients with acute coronary syndromes [[3], [4], [5]]. In our cohort, the observed incidence of CIN is consistent with prior reports from mixed STEMI and NSTEMI populations undergoing urgent revascularization [15,19]. Hemodynamic stress, inflammatory activation, and procedural complexity in acute MI may further increase renal susceptibility in this setting [20].

Baseline renal dysfunction has consistently been identified as one of the most important determinants of CIN [3,21]. In our analysis, chronic kidney disease demonstrated the strongest independent association with CIN, supporting the concept that reduced renal functional reserve represents a key substrate for contrast-related injury. These observations are consistent with current pathophysiological models highlighting the roles of medullary hypoxia, oxidative stress, and endothelial dysfunction in vulnerable kidneys [6,7].

In addition to renal dysfunction, systemic inflammation has been increasingly recognized as a contributor to both acute coronary syndromes and acute kidney injury [8,22]. Acute MI is characterized by substantial inflammatory activation triggered by plaque rupture and ischemia–reperfusion injury [10]. Several studies have evaluated inflammation-derived indices—including NLR, SII, and CRP-based markers—in relation to cardiovascular outcomes and CIN [[11], [12], [13], [14]]. However, most investigations have focused on individual inflammatory parameters.

Recent evidence has further highlighted the prognostic importance of the systemic immune-inflammation index (SII). Bağcı et al. demonstrated that elevated SII independently predicted contrast-induced nephropathy in patients with ST-segment elevation myocardial infarction undergoing primary PCI [23]. Similarly, Yang et al. reported that inflammatory biomarkers were independently associated with contrast-induced acute kidney injury in patients with acute coronary syndrome undergoing PCI [24]. In parallel, recent reviews have emphasized that inflammation plays a central role in the development of contrast-associated acute kidney injury and should be incorporated into contemporary risk assessment strategies [25].

In the present study, isolated inflammatory indices were not consistently higher among patients who developed CIN. Nevertheless, when inflammatory parameters were integrated with renal variables into a composite framework, the resulting score demonstrated an independent association with CIN. This finding suggests that inflammatory activation may contribute to renal vulnerability in a context-dependent manner, particularly when combined with underlying renal susceptibility. Therefore, integrating inflammatory status with baseline renal function may provide a more comprehensive assessment of CIN risk than evaluating either component alone.

The improvement in model discrimination after addition of the composite score was modest. In multifactorial clinical conditions such as CIN, substantial increases in AUC are uncommon once major clinical determinants are included in baseline models [21,26]. Therefore, the potential value of an integrative inflammation–renal approach may lie more in incremental refinement of risk assessment than in large improvements in predictive accuracy.

Another important observation of the present study is the independent association between CIN and in-hospital mortality. Even after adjustment for age and left ventricular function, CIN remained associated with a higher risk of death during hospitalization. This finding is consistent with previous studies linking acute kidney injury with adverse cardiovascular outcomes [4,27]. Although causal inference cannot be established in observational analyses, CIN may reflect systemic vulnerability or the cumulative burden of hemodynamic stress and inflammatory activation in patients with acute MI. From a clinical perspective, early identification of patients at increased risk of CIN using simple bedside tools such as the IRIS score may facilitate timely implementation of preventive strategies, closer monitoring of renal function, and individualized peri-procedural management in accordance with current recommendations for AKI prevention [25,28].

## 4 Limitations

Several limitations should be considered. First, the retrospective single-center design may limit generalizability and introduces the possibility of residual confounding despite multivariable adjustment. Second, although internal validation was performed, external validation in independent cohorts was not available; therefore, the performance of the composite score in other populations remains uncertain. Third, the number of in-hospital deaths was relatively small, restricting the complexity of multivariable modeling for mortality analyses. Fourth, inflammatory markers were measured only at admission, and serial measurements were not available to evaluate dynamic inflammatory changes. Finally, important procedural and clinical variables—including exact contrast volume and type, hydration protocols, vascular access site (radial versus femoral), cardiogenic shock, use of renin–angiotensin–aldosterone system inhibitors or diuretics, and the need for renal replacement therapy—were not consistently available because of the retrospective nature of the study. These factors may have influenced the occurrence of CIN and should be addressed in future prospective multicenter studies.

## 5 Conclusion

In patients with acute myocardial infarction undergoing PCI, contrast-induced nephropathy was common and independently associated with in-hospital mortality. Age and baseline renal dysfunction remained the principal determinants of CIN. An inflammation–renal composite score was independently associated with CIN and may provide additional insight into risk stratification in this population.

These findings underscore the multifactorial nature of CIN and highlight the potential value of integrating systemic inflammatory activity with renal vulnerability in the assessment of contrast-related renal risk. Given that the IRIS score is derived from routinely available clinical and laboratory parameters, it may represent a practical bedside tool for identifying patients at increased risk of CIN before contrast exposure. Larger prospective multicenter studies are warranted to validate these findings and determine the incremental value of the IRIS score in routine clinical practice.

## Informed consent

The requirement for written informed consent was waived due to the retrospective design and the use of anonymized data.

## Ethics approval

The study was approved by the Non-Interventional Clinical Research Ethics Committee of SBÜ Van Training and Research Hospital (Approval No: GOKAEK-2024-02-09,20.12.2024). The study was conducted in accordance with the principles of the Declaration of Helsinki.

## Data availability

The data that support the findings of this study are available from the corresponding author upon reasonable request.

## Declaration of generative AI and AI-assisted technologies in the manuscript preparation process

During the preparation of this work, the authors used AI to assist with English language editing, improvement of academic writing style, and refinement of the manuscript. The authors critically reviewed and edited all AI-generated output and take full responsibility for the content of the published article.

## Funding

This research received no external funding.

## CRediT authorship contribution statement

**Ahmet Ferhat Kaya:** Conceptualization, Data curation, Formal analysis, Investigation, Methodology, Validation, Writing – original draft, Writing – review & editing. **Görkem Ayhan:** Data curation. **Veysi Can:** Data curation. **Emrah Özbek:** Data curation, Formal analysis. **Ömer Kümet:** Data curation, Writing – review & editing.

## Declaration of competing interest

The authors declare that they have no conflict of interest.
